# Supplementary material for: Composite Polyurethane-Polylactide (PUR/PLA) Flexible Filaments for 3D Fused Filament Fabrication (FFF) of Antibacterial Wound Dressings for Skin Regeneration
Source: Materials (Basel). 2021 Oct 13;14(20):6054. doi: 10.3390/ma14206054 (PMC8538761; doi:10.3390/ma14206054)
Supplement: Supplementary file 1 [file materials-14-06054-s001.zip › materials-1386326-supplementary.pdf]

SUPPLEMENTARY DATA TO:

COMPOSITE POLYURETHANE-POLYLACTIDE (PUR/PLA) FLEXIBLE FILAMENTS FOR 3D FUSED FILAMENT FABRICATION (FFF) OF ANTIBACTERIAL WOUND DRESSINGS FOR SKIN REGENERATION

Paweł Szarlej[1], Iga Carayon[1], Przemysław Gnatowski[1], Marta Glinka[2], Anna Brillowska-Dąbrowska[3]*, Justyna Kucińska-Lipka[1]*

1. Gdansk University of Technology, Faculty of Chemistry, Department of Polymer Technology, Narutowicza St. 11/12, 80-233 Gdansk, Poland

2. Gdansk University of Technology, Faculty of Chemistry, Department of Analytical Chemistry, Narutowicza St. 11/12, 80-233 Gdansk, Poland

3. Gdansk University of Technology, Faculty of Chemistry, Department of Molecular Biotechnology and Microbiology, Narutowicza St. 11/12, 80-233 Gdansk, Poland

***Corresponding authors:** iga.carayon@pg.edu.pl, anna.brillowska-dabrowska@pg.edu.pl*

Sample	Brittle fracture	Tensile rupture
TPU	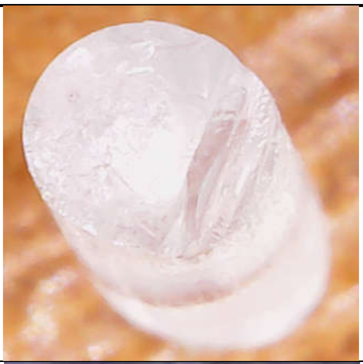	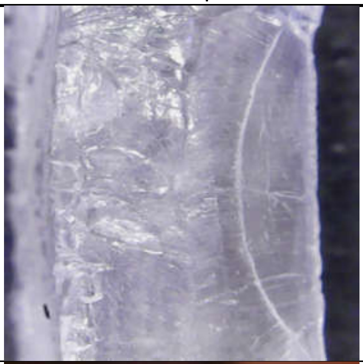
PLA	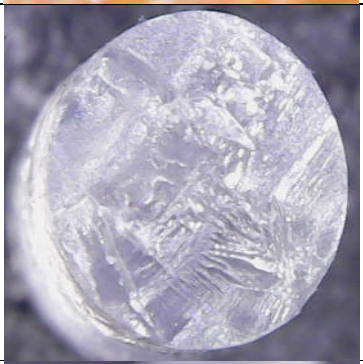	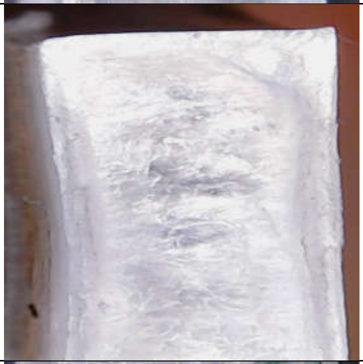
COMP-2,5PLA	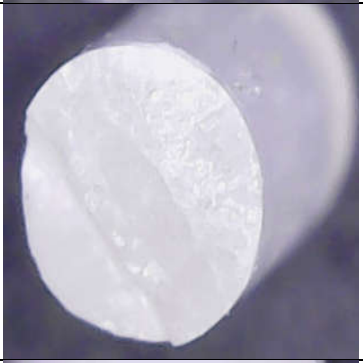	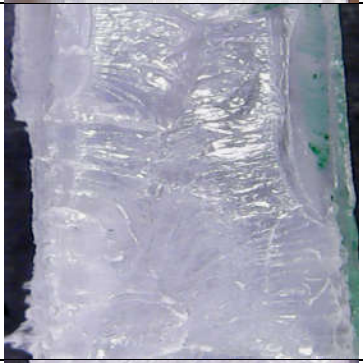
COMP-5PLA	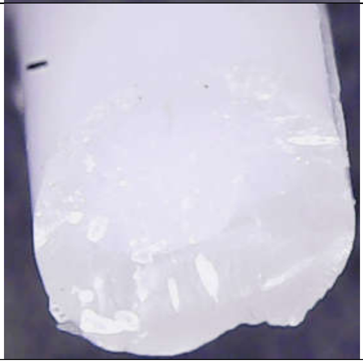	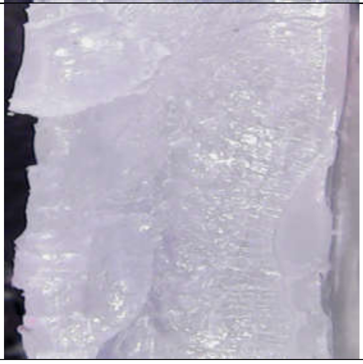

Figure S1. *Optical microscopy of obtained composite filaments*

Sample	Brittle Fracture	Tensile rupture
COMP-7,5PLA	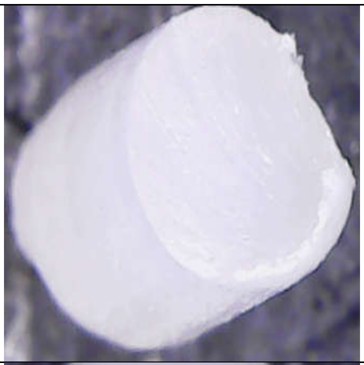	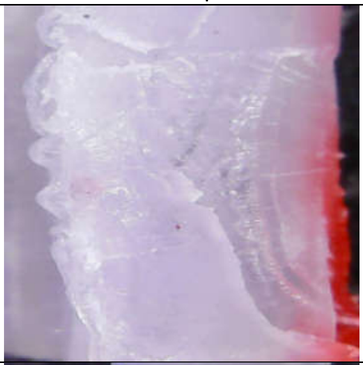
COMP-10PLA	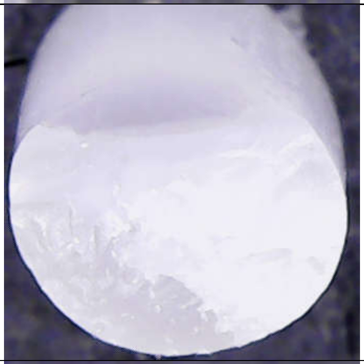	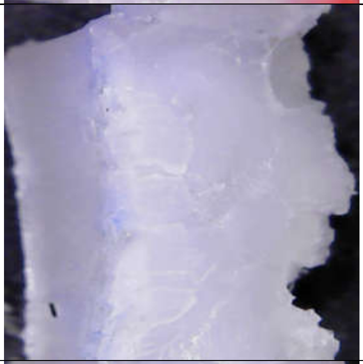
COMP-12,5PLA	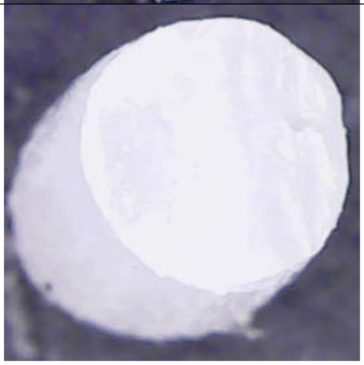	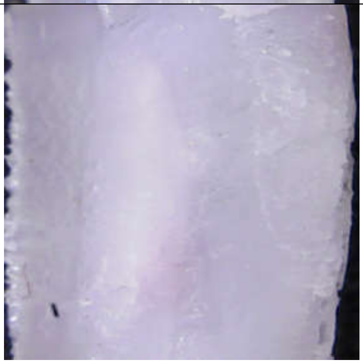

Figure S1. *Brittle fractures and tensile ruptures cd.*

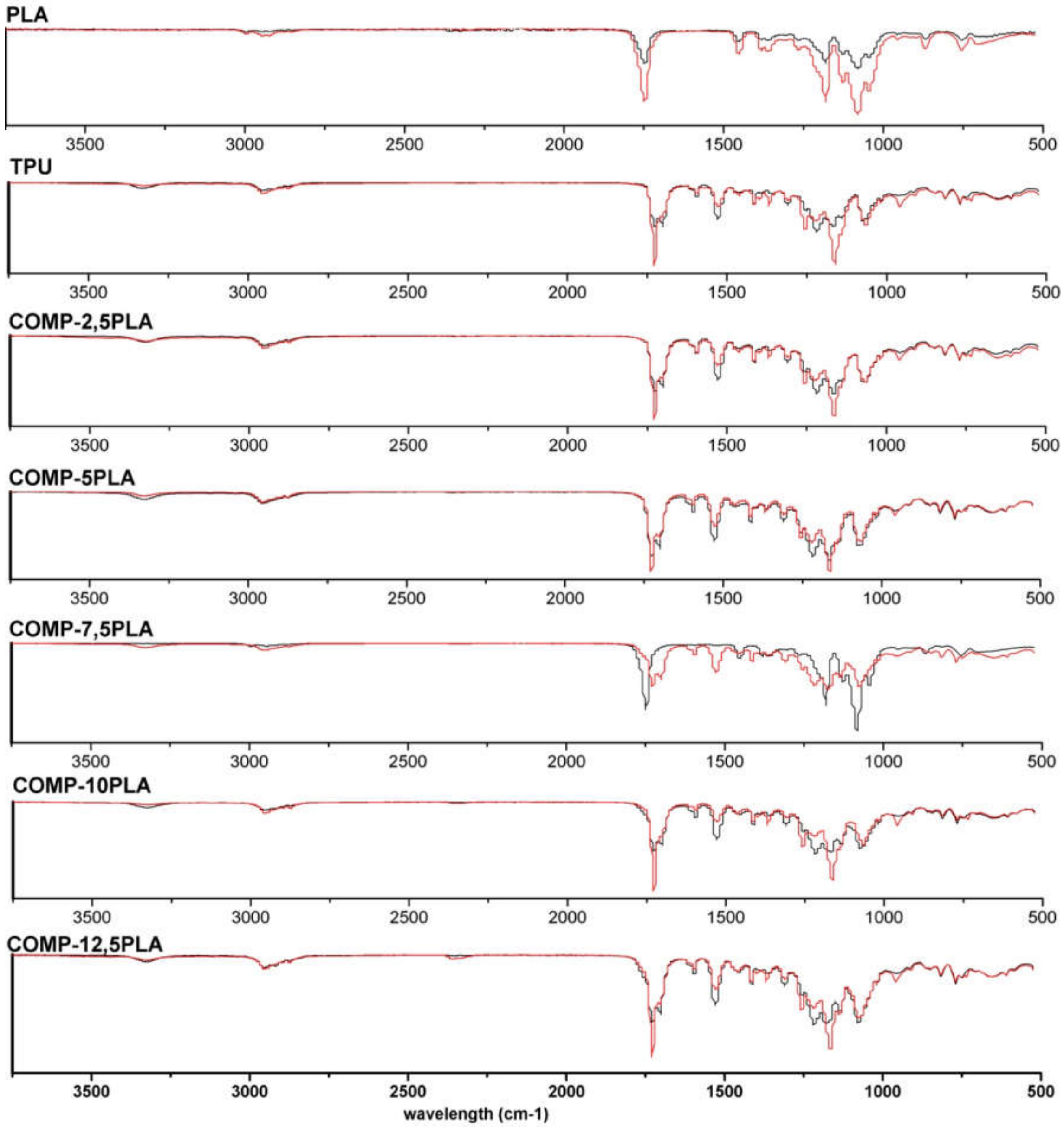

Figure S2. *FTIR spectra before and after degradation in: 2M HCl*

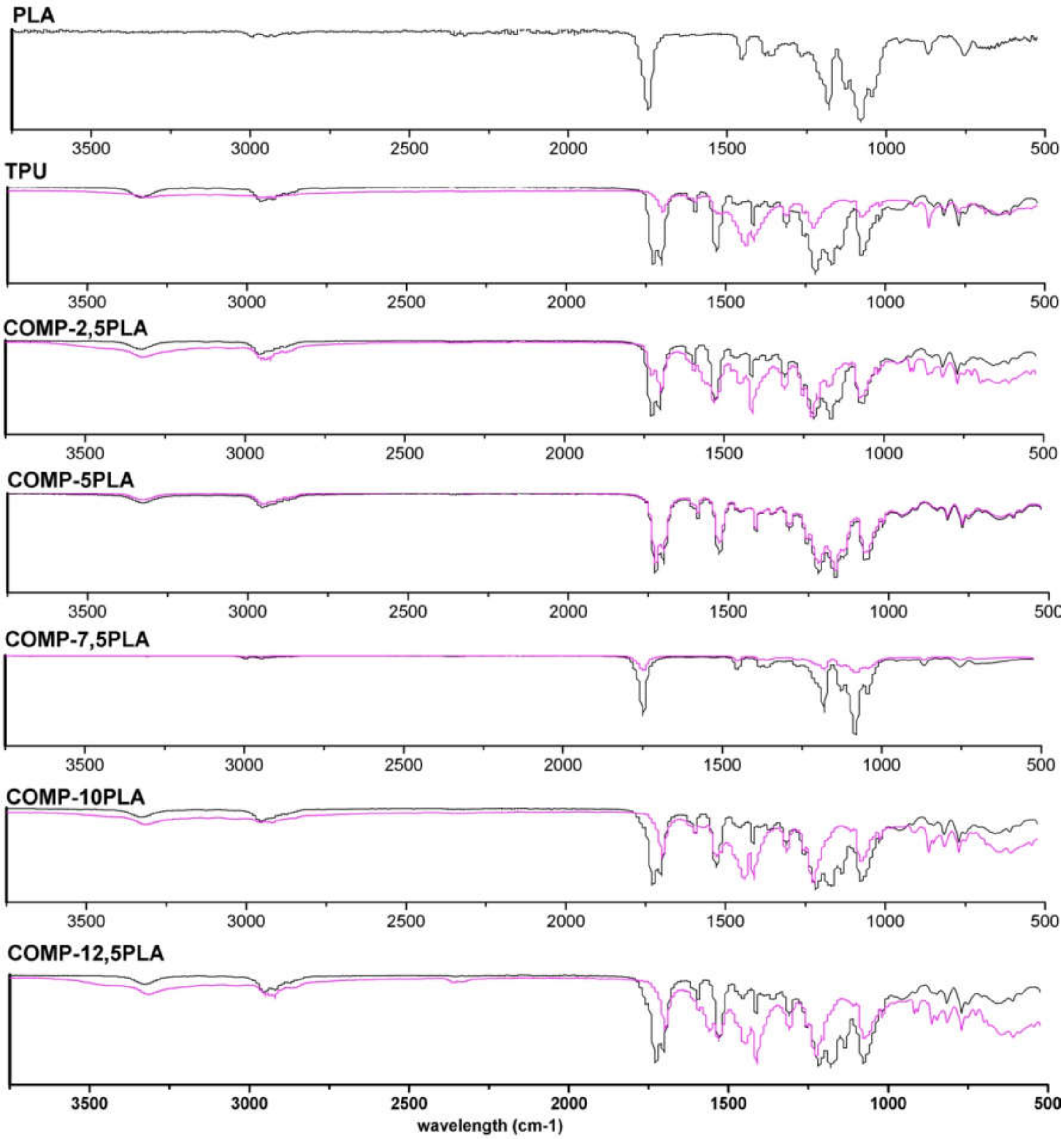

Figure S3. *FTIR spectra before and after degradation in 5M NaOH*

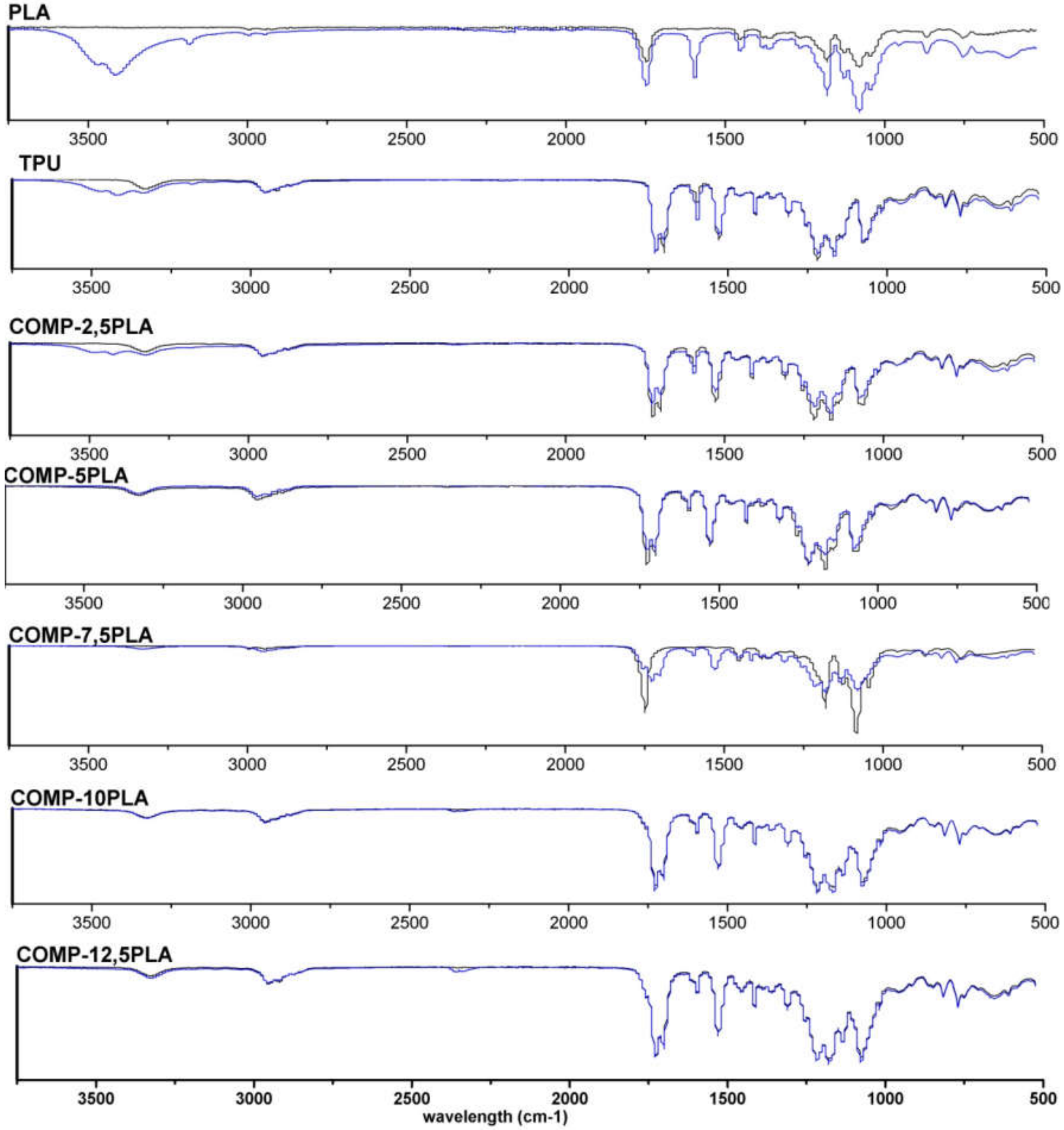

Figure S4. *FTIR spectra before and after degradation in 0,1M $CoCl_2$ in 20% H_2O_2*
